# Prevention in the elderly: A necessary priority for general practitioners

**DOI:** 10.1080/13814788.2017.1350646

**Published:** 2017-08-01

**Authors:** Christos Lionis, Patrik Midlöv

**Affiliations:** ^a^ Clinic of Social and Family Medicine, Faculty of Medicine, University of Crete Crete Greece; ^b^ Center for Primary Health Care Research, Department of Clinical Sciences, Lund University, Skåne University Hospital Malmö Sweden

**Keywords:** Prevention, elderly, health and active ageing, research in general practice

## Abstract

Prevention is viewed as a key issue for general practice, yet there is a lack of evidence regarding general practitioners’ interventions in both middle-aged and elderly people. This is despite the fact that recommendations and key indicators for monitoring the use of clinical preventive strategies aimed at these groups are available and that both the World Health Organization and European Commission endorse the importance of interventions for healthy and active ageing. This paper draws on two keynote presentations given at the 2015 autumn meeting of the European General Practice Research Network (EGPRN) in Edirne, Turkey (17–20 October 2015). According to the EU2020 strategy, general practitioners should design and implement prevention services and programmes to promote healthy and active ageing. Their primary focus should be on interventions on multimorbid patients, either by improving prescribing and adherence to medical plans or by targeting to fall and frailty prevention and vaccination uptake.

KEY MESSAGES

- General practitioners should design and implement preventive services and programmes to promote healthy and active ageing.
- The focus of research should be on interventions to improve prescribing and adherence to medical plans—especially in multimorbid patients—and on personalized health management: fall prevention, vaccination uptake and frailty.

## Introduction

This paper is based on two keynote presentations at the EGPRN autumn meeting in Edirne, Turkey, 17–20 October 2015. It aims to give a summary of these two presentations (CL and PM), to comment on them and make conclusions with some recommendations applicable to general practice. Arising from one presentation (CL) was the intention to highlight the key determinants of active and healthy ageing (AHA) and to discuss challenges and priorities for prevention in elderly within the general practice setting.

Prevention is seen as a magic bullet in healthcare systems across the world and there are many stakeholders with a significant role to play when it comes to prevention and health promotion in the elderly. If this group is to remain healthy, their living conditions should be as supportive as possible. Therefore, ‘prevention’ remains central for national governments and their healthcare policies as well as for scientific organizations that issue guidance for clinical practice.

The World Health Organization (WHO) defines active ageing as ‘the process of optimizing opportunities for health, participation and security to enhance quality of life as people age’ allowing them to ‘realize their potential for physical, social and mental wellbeing throughout the life course’ [1]. Similarly, at the end of 2011, the European Commission (EC) launched the European Innovation Partnership on Active and Healthy Ageing (EIP on AHA) to clearly signal its interest in enhancing interdisciplinary and cross-sectional approaches to tackling demographic ageing and its associated cost and burden [2].

The elderly themselves have a major role to play. Good health requires individuals to be actively aware of how to look after their well-being. A healthy lifestyle is only possible when it is accepted by the individual. For example, the health benefits of exercise and risks of inactivity are well known, but despite these facts, many older adults are physically inactive and this inactivity increases with age [3–5].

Healthcare systems and practitioners play an important role in prevention. Prevention has been prioritized as a key issue in general practice for many years and it is one of the six core characteristics of the European definition of general practice. According to this definition, general practitioners (GPs) should ‘promote health and wellbeing by applying health promotion and disease prevention, cure, care and palliation and rehabilitation’ (Wonca Europe http://www.woncaeurope.org/http://www.woncaeurope.org/). Aligned to the framework of this European definition, the European Network for Prevention and Health Promotion in General Practice (EUROPREV) underlines that evidence-based disease prevention and health promotion should constitute an important part of the daily practice of European GPs to offer high-quality primary care while endorsing certain key statements to primary care as illustrated in Box 1.

Box 1 European network for prevention and health promotion in general practice/family medicine (EUROPREV) 2010. Promoting a prevention strategy. Available from: http://europrev.woncaeurope.org/

**Table:** 

EUROPREV strongly endorses the following statements Evidence-based disease prevention and health promotion should form an important part of the daily practice of European general practitioners to offer high-quality primary care. As far as chronic non-communicable diseases are concerned, general practitioners have a particularly important role in: a. counselling and promoting healthy lifestyles; b. identifying possible health risks in their patients; c. offering interventions to decrease health risks; d. evaluating outcomes. Cost effectiveness, resource prioritization and other logistical factors should be considered at local, national and international levels when implementing preventive activities in clinical practice. Ethical and legal concerns must be resolved before any preventive activity in GP is undertaken. Adult patients and the parents of child patients must be involved as a partner in the planning of preventive activities and also in decision-making as regards the measures needed. A high level of vigilance, such as evidence based, focused on individuals at high risk and rigorous documentation for long-term results and side effects, is required when medications are used to prevent illness in healthy people. General practitioners should be acutely aware of the possible harm that preventive activities may entail. General practitioners should consider equity and accessibility issues in preventive tasks, ensuring these reach those who need them most.

There is a lack of evidence regarding GPs’ interventions both in middle-aged and elderly people, despite policies rich in recommendations and key indicators for monitoring the use of clinical preventive strategies [6]. Although the statements by EUROPREV pinpoint the need for strong prevention in healthy living and healthcare settings, various documents report gaps between GP knowledge and practice including the domains of nutrition and vaccination coverage [7–9].

## Key determinants of active and healthy ageing: the EU2020 strategy

The Joint Research Centre of the European Commission classified the key determinants of active and healthy ageing into six categories (Box 2) [10]. GPs are uniquely placed to recognize the presence of these determinants and their impact on individuals and families, and, to intervene by motivating them to adopt new behaviours.

Box 2 Determinants of active and healthy ageing (Joint Research Centre of the European Commission) [10].

**Table:** 

Determinants	Example
Economic determinants	Income, work and social protection
Health and social service systems	Access to quality health and social services
Physical environment	Housing, neighbourhood and surroundings, transportation
Social environment	Social support and degree of social interaction
Cultural and personal determinants	Cultural values, norms and traditions, genetic influence
Behavioural determinants	Life-style behaviour, well-balanced diet, physical activity, smoking cessation, moderate alcohol consumption, appropriate use of medications

Towards the above direction, the EU worked on a 10-year economic growth strategy; ‘a projected 45% increase in the number of people aged 65 and over in the next 20 years, financing rising healthcare costs and access to a dignified and independent life for the aging population will be central to the political debate.’ It decided to invest in a strategy that aims to keep people healthy and active by giving the health sector a key role in improving skills, creating jobs and positively impacting on people’s productivity and competitiveness [11]. Box 3 highlights the six domains within the EC strategy where the health sector must invest efforts to promote good health [11]. The EC approach offers an excellent foundation where GPs can introduce a discussion on how to broaden its scope and support research initiatives and interventions.

Box 3 The EC 2020: six domains where the health sector has to invest efforts to promote good health [11].

**Table:** 

• Better prescription and adherence to medical plans for older patients• Personalized health management, starting with a Falls Prevention Initiative• Prevention and early diagnosis of frailty and functional decline, both physical and cognitive, in older people• Replicating and tutoring integrated care for chronic diseases, including remote monitoring at regional level• Development of interoperable independent living solutions, including guidelines for business models• Social Innovation for age-friendly buildings, cities and environment

## Challenges and priorities for research on prevention in the elderly

Although they are considered a high priority for GPs, rational prescribing and adherence to treatment always challenge healthcare practitioners. In its document on the action plan for ‘prescription and adherence to treatment,’ the EIP on AHA identified several key gaps for potential future actions [12]. These are grouped into four categories: action on improving patients’ adherence to medications; empowerment of patients and caregivers (with specific attention to the lack of training for GPs to monitor adherence to treatment protocols); research and methodology on ageing issues; and health service improvement issues.

The document relating to research and methodology on ageing issues underlines ‘the lack of identified biomarkers to monitor health status and genetic defects that would eventually lead to frailty and functional decline’ and highlights ‘the lack of a critical mass of competitive interdisciplinary research groups dedicated to translational medicine.’

### A focus on polypharmacy

There is a large body of evidence to show that GPs can, after training, reduce the burden of polypharmacy and recommend appropriate use of over the counter (OTC) medicines [13,14]. Researchers in general practice could borrow knowledge and methodology from social learning theories to modify prescribing behaviour [15]. This is a key message acquired from a FP7 European collaborative project (http://www.otcsociomed.uoc.gr /). A focus on self-management appears to be a challenge for general practice and it is occurring together with new approaches to defining health [16]. A prospective pre-/post-intervention study from Spain presented at the 2015 EGPRN autumn meeting reported an effective reduction of neuroleptic use for the management of behavioural and psychological symptoms in a nursing home [17]. In the Netherlands, a qualitative study explored GPs’ considerations on medication management for complex elderly patients [18]. It suggested that medication reviews, electronic decision-making and insight into the practice’s frail population are promising tools to facilitate management. In addition, another Dutch study presented at the EGPRN autumn meeting studied the medication knowledge of community dwelling older patients with polypharmacy [19]. It concluded that there was little understanding among older patients regarding their drugs and it stressed a need for more attention to people aged 80 years and over.

### A focus on falls

Falls and the subsequent injuries among older people cause a large share of the burden of disease and disability among this group but most falls seem preventable. This is particularly true of falls induced by inadequate pharmacotherapy, which is an important target for GPs [20,21]. There are several other drug-related consequences or problems that are common, costly, and often preventable. In the prevention work of GPs, it is appropriate to minimize at least the harm that inadequate pharmacotherapy may cause. At the 2015 EGPRN autumn meeting, a Spanish RCT reported its first results on the effectiveness of a primary care intervention using the Nintendo TM Wii to improve balance and decrease falls in the elderly [22]. The initial results suggested an improvement on one-foot stationary test and reduction of the fear of failing.

### A focus on frailty

Frailty is strongly associated with multimorbidity and it is also a central issue in the EC strategy and the current recommendations. Multimorbidity and polypharmacy have also received much attention in the current literature and the EC has also discussed these priorities at a meeting in Brussels (December 2015), where an integrated approach was considered to have a positive impact on health system performance and cost effectiveness [14,23–25]. The EIP on AHA has issued an action plan emphasizing that ‘understanding the risk factors for frailty is a prerequisite for implementing programmes for early detection, prevention and management to reduce future demand, improve outcomes and enhance vitality and quality of life’ [10]. It indicates that actions could be undertaken by front-line physicians to develop innovative approaches for screening, identifying and targeting frail older people. Two studies at the 2015 EGPRN autumn meeting focused on frailty [26,27]. Two of the determinants of frailty are malnutrition and sedentary lifestyle. Malnutrition (reduced nutrient intake and/or impaired metabolism) is associated with certain aged-related conditions including changes in mobility, sensory, eye health, oral and gastrointestinal health, cognitive function and several chronic illnesses. The WHO Regional Office for Europe has issued a food and nutrition action plan with a range of objectives to create healthy food and drink environments and promote the benefits of a healthy diet throughout life, especially for the most vulnerable groups [28]. Promoting physical activity in the elderly is also a challenging subject for GPs, while nutritional interventions and physical training in malnourished frail elderly people are receiving growing attention in the literature [29]. A Greek study reported a high prevalence of frailty among people served by a charitable organization in Crete during the austerity period [26]. A Spanish study attempted to evaluate the effectiveness of a multifactorial intervention based on physical activity, diet, memory workshops and medication review [27]. It reported improvements in the prescription rates, short physical performance battery, hand grip strength and cognitive performance.

### A focus on vaccination

The elderly are vulnerable to infectious diseases for many reasons but mostly because they have multiple chronic medical conditions and decreased immune systems, a situation reinforced by an unwillingness to take booster injections against several diseases. This highlights the challenge of increasing vaccination uptake and further research in general practice is required to improve health outcomes by preventing diseases (e.g. community acquired pneumonia, flu, herpes zoster, pertussis, etc.) reducing healthcare cost and protecting the community. It is a relevant issue to the third domain of the EC2020, and the fourth (replicating and tutoring integrated care). Multidisciplinary and integrated care seems to be the cornerstone of promoting healthy ageing, although there are certain European settings where both concepts are yet to be discussed and embedded in national health systems [30].

### 2017 epilogue

A recent search in the PubMed by using ‘prevention programme,’ ‘elderly,’ and ‘general practice’ as search terms identified 59 publications during the period 2015–2017. When the ‘clinical trial’ article type has been activated, the list included nine publications. Four out of nine were relevant to the subject of this article. Rees et al., conveyed no pleasant news when an RCT of a self-management programme for low vision rehabilitation services reported limited benefit of such programmes on vision-specific quality of life [31]. Conversely, Ligthart et al., reported in their qualitative study that the approach of the healthcare provider is crucial to successfully engage old people in long-term preventive consultations [32]. It is a challenging epilogue to an era where GPs could contribute more either to research or clinical practice in the coming years.

## Implications

The selected examples of papers presented at the 2015 EGPRN autumn meeting highlight that there is ample room for preventive interventions in the elderly. Furthermore, the lack of skills in certain primary care settings may impede the implementation of effective interventions. Team-based approaches and skills in behavioural change deserve additional attention [33].

GPs should play an active part in health promotion and prevention in the elderly. They should work collaboratively with secondary care, service providers and other care providers to enhance prevention in older people, focusing on the key determinants of active and healthy ageing as reported by the Joint Research Centre of the European Commission [10]. Evidence-based interventions with a focus on improving prescription and adherence to medical plans especially in multimorbid patients, personalized health management—with a key approach to falls prevention, vaccination uptake, and frailty—need to be designed and implemented. However, both content and methodology of such interventions need to be further discussed and it seems that it is a fertile ground for a future meeting with a focus on research, there is ample room for more substantial research on prevention in older people through the lens of primary care.

## Conclusions

Prevention in the elderly should focus on evidence-based interventions regarding frailty, medication use, personalized health management, falls prevention, and vaccination uptake. Content and methodology of such interventions need to be discussed further.
